# Helicobacter pylori Eradication Using Empirical Therapy in Symptomatic Pediatric Patients: A Multicenter Real-World Study From Colombia

**DOI:** 10.7759/cureus.106089

**Published:** 2026-03-29

**Authors:** José Fernando Vera Chamorro, Carolina Zambrano, Constanza Camargo, Nataly González, Juan Riveros, Andrés Enríquez, Ailim M Carias Domínguez, Michelle Higuera, Diana Quimbayo, César Moreno, Luis Ramírez, Claudia Losada, Diego Blanco, Otto Calderón, Mariana Vásquez

**Affiliations:** 1 School of Medicine, Universidad de los Andes, Bogotá, COL; 2 Department of Pediatric Gastroenterology, Fundación Santa Fe de Bogotá, Bogotá, COL; 3 Department of Gastroenterology, PediAFe Research Group, Bogotá, COL; 4 Department of Gastroenterology, Fundación Santa Fe de Bogotá, Bogotá, COL; 5 Department of Pediatrics, PediAFe Research Group, Bogotá, COL; 6 Department of Gastroenterology, Universidad de los Andes, Bogotá, COL; 7 Department of Gastroenterology, Hospital Infantil Los Angeles, Pasto, COL; 8 Department of Gastroenterology, Hospital Universitario Erasmo Meoz, Cúcuta, COL; 9 Department of Gastroenterology, Universidad El Bosque, Bogotá, COL; 10 Department of Gastroenterology, Unidad de Gastroenterología Pediátrica Dr. Juan Pablo Riveros, Bogotá, COL; 11 Department of Gastroenterology, Hospital Infantil de Manizales, Manizales, COL; 12 Department of Gastroenterology, Hospital Universitario Fundación Valle del Lili, Bogotá, COL; 13 Department of Gastroenterology, Hospital Departamental de Villavicencio, Villavicencio, COL; 14 Department of Gastroenterology, Hospital Federico Lleras Acosta, Ibagué, COL; 15 Department of Gastroenterology, Hospital San Vicente Fundación Medellín, Medellín, COL; 16 Department of Gastroenterology, Clínica Gastroquirúrgica, Cúcuta, COL; 17 Department of Gastroenterology, Clínica Imbanaco Grupo Quirón Salud, Cali, COL; 18 Department of Epidemiology, Fundación Santa Fe de Bogotá, Bogotá, COL; 19 Department of Epidemiology, Subdirección de Estudios Clínicos, Bogotá, COL

**Keywords:** children, colombia, eradication, helicobacter pylori, real-world study

## Abstract

Background

*Helicobacter pylori* (Hp) infection remains a major public health challenge in Colombia, a region with a high incidence of gastric cancer. Optimizing amoxicillin dosing and adherence to weight-based regimens are key modifiable factors to improve eradication.

Objective

This multicenter study aimed to evaluate real-world Hp eradication rates in Colombian children and identify independent predictors of treatment success.

Methods

Secondary analysis of the multicenter “LatamPed-Hp” REDCap registry in REDCap included 26 centers in 14 Colombian cities, including treatment-naïve children with confirmed Hp infection. Demographic data, clinical symptoms, and pharmacological regimens were recorded. Eradication was confirmed after four weeks post-treatment primarily via non-invasive methods. Factors associated with success were analyzed using bivariate tests and a multivariable logistic regression model.

Results

A total of 330 patients were included (mean age 11.0 ± 3.8 years; 52.7% female). The overall eradication rate was 81.8% (270/330). Bivariate analysis showed that success was significantly associated with treatment adherence (p < 0.001), absence of non-gastrointestinal comorbidities (p = 0.005), and adequate amoxicillin dosing (p = 0.017). In the multivariable model, adequate weight-based amoxicillin dosing was a robust independent predictor of success (adjusted odds ratio (aOR) 2.76; 95% confidence interval (CI): 1.11-6.86; p = 0.028). Adherence remained the strongest protective factor (aOR 48.99; p < 0.001), while non-gastrointestinal comorbidities increased the odds of failure (aOR 0.21; p < 0.001). The model demonstrated high accuracy (84.59%) and sensitivity (98.31%).

Conclusion

Eradication rates in Colombian children remain below international targets. Pharmacological precision, specifically adequate amoxicillin dosing, is a critical modifiable factor for success. Ensuring strict adherence to weight-based protocols and improving treatment compliance are essential strategies to optimize Hp eradication and reduce long-term gastric cancer risk in this population.

## Introduction

*Helicobacter pylori* (Hp) is a major human pathogen that colonizes the gastric epithelium and is critically associated with chronic gastritis, peptic ulcer disease, mucosa-associated lymphoid tissue (MALT) lymphoma, and gastric adenocarcinoma [1,2]. Consequently, the World Health Organization (WHO) classifies Hp as a Group I carcinogen [3]. Globally, the prevalence of Hp infection in children exhibits significant geographic disparity, reaching up to 80% in developing nations compared to <15% in industrialized countries [2,4]. In Colombia, reported pediatric prevalence ranges from 30% to 78% [5], where factors such as overcrowding, limited access to clean water, poor household sanitation, and low parental education remain major risk factors for early infection [6,7]. Furthermore, reinfection is frequent in children under five years old, particularly in low-income environments [8].

High infection rates during childhood have been linked to a sixfold increase in the lifetime risk of gastric cancer. This underscores the necessity for early and effective eradication strategies, especially considering that gastric cancer is the leading cause of cancer-related mortality in Colombia [9,10]. In Latin America, eradication rates using standard triple therapy have ranged from 64% to 75%, varying by treatment duration and local antibiotic resistance patterns [11]. While sequential therapy has achieved eradication rates above 80% in some studies and susceptibility-guided regimens can reach up to 98% [12-14], treatment adherence remains a critical determinant of success, as suboptimal compliance markedly decreases efficacy [4]. Although international guidelines recommend post-treatment confirmation of eradication-via stool antigen or urea breath test-at least four weeks after therapy completion, access to these diagnostic tools and the associated costs remain major barriers to systematic follow-up across Latin America [15,16].

Despite the high burden of gastric disease and variable antibiotic resistance in the region, few multicenter studies have examined the determinants of eradication in Latin American and Colombian children [4,5,7,17]. While clarithromycin remains a viable first-line antibiotic due to historically low resistance rates reported in Colombian pediatric populations, significant uncertainty persists regarding its real-world efficacy [5,7].

To address these gaps, the present study sought to evaluate the clinical outcomes of Hp management in a multicenter cohort from one country (Colombia). The primary objective was to determine the real-world eradication rate in symptomatic pediatric patients across various Colombian centers using empirical first-line therapy as per the 2017 NASPGHAN/ESPGHAN/LASPGHAN guidelines. The secondary objectives were to identify the clinical and pharmacological predictors of eradication failure, specifically evaluating the impact of treatment adherence, and the accuracy of weight-adjusted antibiotic dosing.

## Materials and methods

Study design and population

A secondary analysis was conducted using data from the multicenter LatamPed-HP registry, hosted on the REDCap platform, specifically focusing on Colombian participants enrolled between January 2018 and June 2024. The study included children and adolescents under 18 years of age presenting with gastrointestinal symptoms and confirmed Hp infection. In strict alignment with international pediatric guidelines, diagnostic testing was not performed as a "test-and-treat" screening tool for functional pain. Instead, all participants (100%) underwent upper gastrointestinal endoscopy based on specific clinical indications, such as persistent epigastric pain or suspected peptic ulcer disease. Patients who had received eradication therapy within the previous year or who had used antibiotics, proton pump inhibitors (PPIs), or H_2_ receptor antagonists in the month prior to diagnosis were excluded.

Diagnostic procedures

The diagnosis of Hp infection was established through invasive methods during endoscopy in 100% of the study population. In accordance with the Sydney System, a minimum of five gastric biopsies were collected from each patient. Histopathological evaluation was the most frequent diagnostic method utilizing hematoxylin and eosin (H&E) staining. The rapid urease test (RUT) was performed using a commercial urea-agar kit, with results interpreted based on colorimetric changes within 24 hours. Bacterial culture was performed when available. The combination of two or more invasive methods was accomplished in a few of the 26 participating Colombian centers.

Treatment protocols and dosing accuracy

Patients were treated with empirical first-line therapies as per the 2017 NASPGHAN/ESPGHAN/LASPGHAN guidelines. The regimens included standard triple therapy (PPI + amoxicillin + clarithromycin), alternative triple therapy (PPI + amoxicillin + metronidazole), and quadruple therapies with or without bismuth, all prescribed for a 14-day duration. Dosing accuracy was defined as the administration of antibiotics and PPIs according to the weight-bracket dosages recommended by international guidelines. Suboptimal dosing was specifically analyzed as an independent predictor of treatment outcome.

Outcome assessment: eradication and adherence

Eradication success was defined as the absence of infection confirmed at least four weeks after completing treatment. Non-invasive testing was the predominant confirmatory method, including the urea breath test and monoclonal stool antigen tests. Invasive follow-up via endoscopy was reserved for a high-risk subgroup of cases with persistent clinical symptoms or prior complicated peptic disease requiring direct mucosal visualization.

Treatment adherence was defined as the completion of at least 80% of the prescribed regimen. This was assessed during follow-up visits using a standardized hetero-administered questionnaire, where parents or caregivers provided a self-report on the completion of the antibiotic course and PPIs. Eradication failure was documented when post-treatment tests remained positive. Adverse events were classified by gastrointestinal symptoms experienced during therapy, such as epigastric pain, diarrhea, and nausea.

Statistical analysis

Continuous variables are presented as mean (standard deviation) or median (interquartile range), while categorical variables are expressed as absolute and relative frequencies. Bivariate analyses were performed to explore associations with eradication. Candidate variables-selected based on clinical relevance or a p < 0.20 in bivariate screening-were entered into a multivariable logistic regression model. To address potential confounders such as adherence bias and dosing accuracy, these factors were tested as independent predictors. We utilized a backward elimination approach to derive a parsimonious final model. The final adjusted model reported adjusted odds ratios (aORs) with 95% confidence intervals (CIs). All analyses were performed using Stata version 19.0 (StataCorp, College Station, TX, USA).

For descriptive and bivariate analyses, a complete-case analysis approach was initially adopted; however, to maximize the use of available information, denominators were adjusted based on the total number of valid observations for each specific variable. In the multivariable logistic regression model, 38 observations were excluded due to missing values in key covariates (primarily adherence and specific dosing accuracy) or the identification of complete separation, resulting in a final analytic sample of n = 292. No imputation methods were applied, as the proportion of missing data for the primary outcome (eradication status) was zero, and for secondary covariates, it remained below 10% of the total cohort.

## Results

A total of 330 eligible patients were identified and included in the final analysis. One hundred seventy-four (52.6%) were female, with a mean age of 11.0 ± 3.8 years. The majority of the cohort belonged to the middle socioeconomic level (n = 197, 60.2%) (Table 1). Abdominal pain was the primary indication for endoscopy (n = 298, 90.3%), with associated symptoms including nausea (n = 146, 44.2%), food aversion (n = 55, 16.7%), dysphagia (n = 38, 11.5%), chest pain (n = 16, 4.9%), hematemesis (n = 13, 4.0%), and melena (n = 1, 0.3%).

**Table 1:** Demographic and clinical characteristics of the study population

Variable	No.	%
Sex		
Female	172	52.6
Male	155	47.4
Age		
Preschoolers	35	10.6
School-age	132	40.0
Adolescents	163	49.4
Socioeconomic status		
High	43	13.1
Middle	197	60.2
Low	87	26.6
Comorbidities		
Functional gastrointestinal disorders	80	24.2
Allergic diseases	32	9.7
Immune-mediated diseases	3	0.9
Family history		
*Helicobacter pylori* infection	75	23.0
Gastric cancer	35	10.7

Comorbidities were assessed and classified as functional gastrointestinal disorders (gastroesophageal reflux disease, constipation, functional abdominal pain, and irritable bowel syndrome), immune-mediated diseases (eosinophilic esophagitis, inflammatory bowel disease, and celiac disease), and allergic diseases (asthma, rhinitis, and dermatitis). Family history of Hp infection and gastric cancer, first and second degrees, was also reported (Table 1).

In alignment with international recommendations, all patients (100%) underwent invasive diagnostic procedures. The most prevalent endoscopic finding was nodularity, followed by gastric or duodenal erosions and peptic ulcers (Table 2). Histopathology was the most utilized diagnostic test, while 76/330 patients were diagnosed using a combination of two or more methods, including the RUT and bacterial culture (Table 2).

**Table 2:** Endoscopic findings and diagnostic and eradication methods for Helicobacter pylori PCR: polymerase chain reaction

	n	%
Endoscopic findings		
Nodularity	260	78.8
Erosions	130	39.4
Ulcers	24	7.3
Diagnostic method		
Histology	324	98.2
Rapid urease test	71	21.5
PCR	0	0
Culture	7	2.1
Combination of ≥2 tests	76	23.0
Methods used for eradication confirmation		
Urea breath test	205	62.1
Stool antigen test	77	23.3
Endoscopy with biopsy	54	16.7

Regarding therapeutic regimens, the majority of patients received standard triple therapy. Alternative triple therapy was used in less than 10% of the cases; quadruple regimens-with or without bismuth-accounted for less than 4% each. A subset of 6.97% patients received non-conventional or incomplete regimens (Table 3). Sequential therapy was not employed in any case, reflecting its limited use in pediatric clinical practice due to its complexity and potential negative impact on treatment adherence.

**Table 3:** Pharmacologic regimens used for Helicobacter pylori eradication *Other non-conventional or incomplete regimens included dual therapies, PPI monotherapy, or combinations lacking a PPI, reflecting the inherent variability of real-world clinical practice despite established international guideline recommendations PPI: proton pump inhibitor

Regimen category	Drug combination	Frequency (n)	Percentage (%)
Standard triple therapy	PPI + amoxicillin + clarithromycin	260	78.8
Alternative triple therapy	PPI + amoxicillin + metronidazole	26	7.9
Bismuth quadruple therapy	PPI + amoxicillin + clarithromycin + bismuth	11	3.3
Non-bismuth quadruple therapy	PPI + amoxicillin + clarithromycin + metronidazole	10	3.0
Other regimens	Incomplete or non-conventional regimens*	23	7.0
Total		330	100

**Figure 1:**
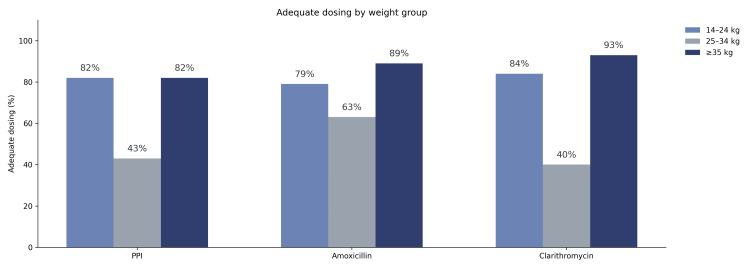
Adequate dosing (%) by drug and weight group PPI: proton pump inhibitor

Dosing accuracy was evaluated according to the weight-based categories recommended by the 2017 ESPGHAN/NASPGHAN guidelines. Suboptimal dosing was most frequent in the 25-34 kg weight group, particularly concerning amoxicillin administration (Figure 1). 

In this study, 299 patients (96.1%) self-reported adherence to the 14-day eradication regimen. The main reasons for non-adherence included difficulties administering the medication, adverse events such as epigastric pain and diarrhea, and administrative barriers. A total of 126 adverse events were reported, most commonly nausea or vomiting (n = 54, 16.4%), diarrhea (n = 28, 8.5%), anorexia (n = 23, 7.0%), headache (n = 17, 5.2%), and, rarely, constipation (n = 1, 0.3%); however, these symptoms led to permanent discontinuation in only a small subset of non-adherent patients. Administration challenges were largely attributed to poor palatability and, in younger children, the required suspension volume. Among non-adherent cases, systemic barriers primarily involved delays in medication authorization or delivery by the insurance provider (EPS), whereas personal barriers reflected caregiver-related logistical interruptions.

The overall Hp eradication rate was 81.8% (270/330). Success was confirmed primarily through non-invasive methods, including the urea breath test (n = 208, 62.5%) and stool antigen test (n = 77, 23.3%), while endoscopy with biopsy was used when non-invasive methods were unavailable (16.4%).

In the bivariate analysis, eradication rates did not differ significantly by sex, age group, socioeconomic status, or the presence of functional gastrointestinal disorders. Similarly, no significant association was found regarding a family history of Hp infection, although a family history of gastric cancer showed a non-significant trend toward lower eradication success (OR = 0.51; 95% CI: 0.23-1.12; p = 0.093).

In contrast, several key factors were identified as significant predictors of treatment outcomes. Adequate amoxicillin dosing was a significant predictor of success (OR = 2.07; 95% CI: 1.14-3.77; χ² = 5.44; p = 0.017), demonstrating that patients receiving the correct weight-based dose achieved higher eradication rates than those who were under-dosed. Furthermore, the presence of non-gastrointestinal comorbidities was negatively associated with eradication (OR = 0.44; 95% CI: 0.24-0.77; p = 0.003). Finally, treatment adherence showed a definitive association with eradication status (Fisher’s exact test, p < 0.001); among adherent patients, the eradication rate reached 83.6%.

To identify independent predictors of treatment success, a multivariable logistic regression model was estimated (n = 292) (Table 4). After adjusting for potential confounders, including sex, age, and socioeconomic status, adequate amoxicillin dosing emerged as a robust independent predictor of eradication success (aOR 2.76; 95% CI: 1.11-6.86; p = 0.028). Additionally, treatment adherence was the strongest protective factor, being associated with markedly higher odds of success (aOR 48.99; 95% CI: 8.37-286.6; p < 0.001). Conversely, the presence of non-gastrointestinal comorbidities was independently associated with a lower likelihood of success (aOR 0.21; 95% CI: 0.09-0.47; p < 0.001). Using a 0.5 probability threshold, the final model correctly classified 83.77% of observations. Other clinical variables, including sex (p = 0.074) and family history of gastric cancer (p = 0.124), were not independently associated with the outcome after adjustment.

**Table 4:** Multivariable logistic regression for eradication of Helicobacter pylori in children (n = 292) The outcome variable was eradication (eradication = 1). OR > 1 indicates higher odds of eradication; OR < 1 indicates lower odds of eradication OR: odds ratio; CI: confidence interval

Predictor	Adjusted OR (aOR)	95% CI	p-value
Non-gastrointestinal comorbidities	0.22	0.09-0.48	<0.001
Adequate amoxicillin dosing	2.76	1.11-6.86	<0.001
Adherence	48.9	8.37-286.6	0.028

The model demonstrated an improved fit compared with the null model and showed no evidence of lack of fit according to the Pearson goodness-of-fit test (χ² = 222.52; p = 0.40). Furthermore, the mean variance inflation factor (VIF) of 1.94 confirmed the absence of significant multicollinearity. Thirty-eight observations were excluded due to missing data in the adherence variable and due to the identification of complete separation (perfect prediction) at one level of the PPI variable. Consequently, the final model was estimated with n = 292.

The model showed improved fit compared with the null model (LR χ²(3)=37.31, p<0.001), with a McFadden pseudo R² of 0.1998. The Hosmer-Lemeshow goodness-of-fit test showed no evidence of lack of fit (p=0.6189). Using a 0.5 probability threshold, the model correctly classified 84.59% of observations (247/292). The model showed high sensitivity (98.31%) but low specificity (25.45%), indicating that it is very effective at identifying cases of eradication but performs poorly in correctly identifying non-eradication cases.

## Discussion

This is the first Colombian multicenter study to evaluate real-world Hp eradication in treatment-naïve children across 26 centers and 14 cities. The observed overall eradication rate was 81.8% (270/330). While this rate is higher than those reported in other developing regions, it still falls below the 90% success target recommended by the ESPGHAN/NASPGHAN and LASPGHAN guidelines [1,18]. Comparative data globally show significant disparity; for instance, eradication rates as low as 26.7% have been reported in Romania due to high clarithromycin resistance [19], and only 38% in Chile using empirical standard therapy [11]. Our results align more closely with multicenter cohorts in the United Arab Emirates (68%) [20] and Latin American adult studies (up to 64%) [21].

A pivotal finding of this study, reinforced by our multivariable analysis, is the impact of pharmacological precision. Adequate amoxicillin dosing emerged as a robust independent predictor of success (aOR 2.76; 95% CI: 1.11-6.86; p = 0.028). Although international guidelines emphasize strict weight-bracket dosing [1,18], this factor is rarely quantified as a predictor in clinical trials. Our data revealed a critical "dosing gap" in children weighing 25-34 kg, where correct dosing rates for amoxicillin and PPIs dropped to between 40% and 63%. The finding that under-dosing significantly increases the odds of eradication failure suggests that therapeutic success in our region is driven not only by biological resistance but also by modifiable provider-related factors.

Regarding antimicrobial resistance, the 18.2% failure rate observed here may reflect evolving resistance patterns. A recent systematic review by Cabrera et al. noted that clarithromycin resistance in Latin America varies widely (8.1% to 79.6%) [22], while a previous Colombian pediatric study suggested a relatively low resistance rate of 8.1% [5]. In our adjusted model (n = 292), treatment adherence was the strongest protective factor (aOR 48.99 for success; p < 0.001), consistent with international trials where optimized adherence is linked to rates exceeding 85% [23]. Conversely, the presence of non-gastrointestinal comorbidities was independently associated with lower success rates (aOR 0.21; p < 0.001), potentially due to complex drug interactions or polypharmacy-related interference with gastric acid suppression.

Consistent with established literature, factors such as sex, age, and socioeconomic status did not independently influence eradication outcomes [4,24]. Although a family history of gastric cancer showed a non-significant trend toward lower eradication in the bivariate analysis (p = 0.093), it was not retained in the final multivariable model. Nevertheless, this remains a clinically relevant subgroup given that early Hp eradication is a primary preventive strategy for gastric malignancy, the leading cause of cancer mortality in Colombia [9,10,25].

Strengths and limitations

The primary strength of this study is its multicenter design, providing a real-world snapshot of pediatric Hp management across diverse Colombian clinical settings. However, we acknowledge several limitations. First, the low rate of bacterial cultures (2.1%) prevents a direct correlation between treatment failure and phenotypic antimicrobial resistance. Second, reliance on self-reported adherence may introduce social desirability bias, potentially overestimating true compliance and therefore adherence association with successful eradication.

Finally, while our multivariable model demonstrated a strong goodness of fit (Hosmer-Lemeshow p = 0.6189) and a high overall accuracy (84.59%), it showed high sensitivity (98.31%) but lower specificity (25.45%). This suggests the model is highly effective at identifying factors that ensure success but less precise in predicting individual failure. This "optimistic" bias is common in populations with high success rates and suggests that unmeasured biological variables-such as CYP2C19 genetic polymorphisms affecting PPI metabolism, specific bacterial virulence factors, or antimicrobial resistance-may further influence non-eradication cases [26].

Future research directions

Future prospective studies should prioritize regional antibiotic susceptibility mapping to facilitate the transition from empirical to susceptibility-guided therapy. In the interim, our findings suggest that clinical outcomes in Colombia can be significantly improved by ensuring strict adherence to weight-based dosing protocols, particularly for amoxicillin. Evaluating the efficacy of optimized high-dose dual therapies or bismuth-based quadruple regimens in the Colombian pediatric population remains a priority for subsequent clinical trials.

## Conclusions

In this multicenter study, the Hp eradication rate among Colombian pediatric patients was 81.8%, a figure that remains below the international quality benchmark. Our analysis identifies pharmacological precision as a critical and modifiable determinant of treatment success. Specifically, adequate weight-based amoxicillin dosing emerged as a robust independent predictor of eradication, while a significant "dosing gap" was observed in children weighing 25-34 kg. These findings suggest that subtherapeutic exposure is a primary driver of eradication failure in our clinical setting.

To improve clinical outcomes in regions with a high burden of gastric cancer, such as Colombia, it is imperative to transition from traditional empirical therapy to protocols ensuring strict adherence to weight-bracket dosing. While the study's multivariable model proved highly effective at identifying factors that ensure success-with a notable 84.59% accuracy and 98.31% sensitivity-the lower specificity highlights the need for future research into unmeasured biological variables, including host genetic polymorphisms and local antibiotic susceptibility mapping. Optimizing current regimens through precise dosing and enhanced treatment adherence remains the most viable immediate strategy to reduce the long-term oncological risks associated with early Hp infection.
